# The Relationship Between Teacher Job Stress and Burnout: A Moderated Mediation Model

**DOI:** 10.3389/fpsyg.2021.784243

**Published:** 2022-01-25

**Authors:** Weiguo Zhao, Xiangrui Liao, Qingtian Li, Wenning Jiang, Wen Ding

**Affiliations:** School of Psychology, Shandong Normal University, Jinan, China

**Keywords:** job burnout, job stress, work–family conflict, self-efficacy, teacher

## Abstract

This study explored the relationship between teacher job stress and job burnout using a sample of 558 primary and secondary school teachers, who were administered with a teacher job stress scale, teacher job burnout scale, work–family conflict questionnaire, and general self-efficacy scale. The results showed that: (1) job stress had a significant predictive effect on work–family conflict and job burnout; (2) work–family conflict played a mediating role in the relationship between job stress and job burnout; (3) self-efficacy was found to play a moderating role in work–family conflict and job burnout. However, this indirect effect was stronger for teachers with high self-efficacy, which means that the protective effects of self-efficacy were limited. These findings add to research on the relationship between teacher job stress and job burnout, and provide ideas for teachers to balance work–family relationships and reduce job burnout.

## Introduction

Teacher job burnout refers to the emotional and behavioral exhaustion caused by the long hours and high-intensity nature of the daily teaching process (Cherniss, 1980; Wu et al., 2016). It consists of three components referred to as emotional exhaustion, reduced personal accomplishment, and depersonalization (Maslach et al., 2001). Teachers have higher levels of psychological stress and burnout compared with other occupations (Kovess-Masfety et al., 2007; Ouellette et al., 2018; Fathi et al., 2021). Job burnout not only directly affects the teaching quality and physical and mental health of teachers (Domitrovich et al., 2016; Capone and Petrillo, 2018), but also has many negative effects on the academic achievement and social behavior of students (Klusmann et al., 2016; Madigan and Kim, 2021). Therefore, exploration of the generation mechanism and influencing factors of job burnout can not only improve mental health problems of teachers, but also indirectly promote mental health and academic progress of students.

According to the conservation of resources theory, individuals with abundant resources can acquire more resources and are less vulnerable to resource loss. On the contrary, individuals reduce resource input due to the pressure caused by lack of resources, resulting in the loss of resources (Hobfoll, 1989). In daily work, primary and secondary school teachers undertake a heavy workload of teaching activities and student management activities, and thus need to invest many internal and external resources (Zhou and Ning, 2020). When teachers face pressure from numerous aspects, such as workload, examination pressure, and student management, it is likely to result in a sense of powerlessness and even frustration due to the limited resources. Previous studies have indicated that there are positive relationships between the occupational stress and teacher burnout (Kosir et al., 2015; Bottiani et al., 2019). However, there is a specific source of stress in Chinese primary and secondary schools, different from the occupational stress of western teachers. For example, examination and career expectations are stressors in the Chinese context. Examination stress refers to the examination scores of students affecting the performance evaluations of teachers. Self-development needs stress refers to, for example, the need for teachers to continually learn new teaching content and working methods to align with the reform of the curriculum and educational evaluation system in China (Zhu et al., 2002; Li et al., 2011). Therefore, it is necessary to investigate the job stress of primary and secondary school teachers based on the particularity of the Chinese cultural background.

Several studies in western countries have suggested certain mediating variables between occupational stress and burnout. For example, psychological capital was found to play a mediating role between job stress and burnout (Kim and Kweon, 2020). However, all these studies have been based on the perspective of individual traits. Individual–context interaction theory points out that individual development can be affected by the interaction between individual trait factors and context factors (Lerner et al., 2006). Therefore, to clarify the development mechanism of job burnout, it is necessary to investigate the mediating and moderating mechanisms of emotional factors (job stress), environmental factors (work–family conflict), and personality traits (self-efficacy) on job burnout from the perspective of multi-factor integration.

The rapid development of the Chinese economy has meant that professional competition has become more intense. People need to spend more time at work than ever before, which undoubtedly takes away time allocated for rest and spending time with their families. This makes it difficult to maintain a balance between family and work, and gives rise to more conflict between family and work (Li et al., 2015). In addition, influenced by the Chinese “family standard” culture, family assumes primary importance in the minds of people (Gao, 2020). Influenced by this culture, teachers have to constantly make choices between work and family. When faced with such conflict and contradictions, the personal resources loss of teachers will accelerate and their emotional exhaustion will intensify (Ji and Yue, 2020). However, few empirical studies have been conducted on the role of work–family conflict in the relationship between job stress and burnout. Hence, the impact of the family environment on teacher burnout is worth investigating.

Moreover, according to the conservation of resources theory, the psychological resources of individuals have an important impact on burnout (Hobfoll and Freedy, 2018). As an important psychological resource, self-efficacy can help individuals to reduce their internal resource loss in the face of external stress, thus alleviating the burnout. A previous study of firefighter burnout has found that self-efficacy can moderate the relationship between perceived stress and burnout, and those with high self-efficacy exhibited less burnout in the face of stress (Makara-Studzinska et al., 2019). However, few studies have investigated the moderating effect of self-efficacy between job stress and teacher burnout and between work–family conflict and burnout; therefore, it is necessary to examine the role of self-efficacy in the mechanism of teacher burnout generation.

To sum up, still there is still gap in knowledge regarding the impact mechanism of job stress on job burnout among the Chinese teachers. This study intends to select primary and secondary school teachers, a group with serious job stress, to investigate the impact of primary and secondary school job stress on burnout, the mediating role of work–family conflict between them, and the moderating role of self-efficacy in the mediating path. This is of great significance for alleviating the job stress of primary and secondary school teachers and improving their level of burnout.

### Teacher Job Stress and Job Burnout

Teacher job stress refers to the unpleasant negative emotional experience of teachers that can lead to excessive physical and mental fatigue, nervous tension, frustration, or distress due to factors, such as excessive working hours, heavy workload, and serious misconduct of students (Roeser et al., 2013). Research studies have indicated that teaching is one of the most stressful occupations (Johnson et al., 2005; Herman et al., 2020; Greenier et al., 2021).

With the educational reform and changes over time, the sources of job stress among primary and secondary school teachers are also changing (Li et al., 2021). First, teachers need to change their previous teaching content and working methods according to the requirements of national policies, which undoubtedly increase their workload. Second, teachers face the stress of school assessment of their teaching quality and performance evaluation. In addition, primary and secondary school teachers face self-development stress, such as poor salaries and limited development prospects. The stress faced due to many aspects, coupled with the problem that their professional effort is disproportionate to the reward received, results in more serious job stress among the primary and secondary school teachers (Qi et al., 2014; Zhou and Ning, 2020). Therefore, there is an urgent need to investigate the current situation of Chinese teacher work stress and the possible impact of such severe work stress on teacher burnout.

The conservation of resources theory suggests that individuals will feel nervous when they face threats, such as excessively high job demands, resources loss, and disproportionate effort and reward of resources (Hobfoll, 1989). Previous research studies revealed that job stress had a positive predictive effect on job burnout (Kosir et al., 2015; Bottiani et al., 2019), and was significantly negatively correlated with job satisfaction (Ouellette et al., 2018). Based on these, we established our first hypothesis:

**Hypothesis 1:** Job stress can significantly positively predict job burnout.

### The Mediating Role of Work–Family Conflict

Work–family conflict refers to the role conflict of internal resources, emotions, and behaviors due to the role pressure at work and in the family, which is multi-dimensional and multi-level. It can be divided into two types: work interfering with family caused by work requirements and family interfering with work caused by family requirements (Frone et al., 1997; Wu et al., 2009). According to spillover theory, the boundary between the work and family contexts is permeable. An individual’s perceived threat of resource loss caused by work pressure can be easily transferred to the non-work context. Therefore, the problems encountered by teachers at work are likely to overflow into the family context (Staines, 1980; Richter et al., 2015). Previous studies on other groups have also revealed that there is a stable connection between job stress and work–family conflict (Ismail and Gali, 2017). Pressure on individuals from work will spread to their spouses and children, and intensify the conflict between work and family (Carlson et al., 2018; Nomaguchi and Fettro, 2019).

In addition, many studies have shown that the conflict between work and family makes it difficult for individuals to deal with the dual roles of work and family, eventually resulting in job burnout (Pu et al., 2017; Ji and Yue, 2020). According to the conservation of resources theory, sufficient resources are the key factor to meet continuous work needs and prevent burnout (Hobfoll and Freedy, 2018). For teachers, family and work can be regarded as the important individual resources, but if there are conflicts between work and family, these can also be regarded as an important source of pressure that negatively affects the balance between work and family (Lambert et al., 2002). This pressure consumes individual psychological resources and promotes job burnout (Simães et al., 2021). Based on this, we established our second hypothesis:

**Hypothesis 2:** Work–family conflict plays a mediating role in job stress and burnout.

### The Moderating Role of Self-Efficacy

It is not necessary that all individuals who experience job stress and work–family conflict will suffer negative adaptation and job burnout. The conservation of resources theory shows that an individual evaluation and response to stressors depends not only on the stressors themselves, but also on the psychological characteristics of an individual (Grandey and Cropanzano, 1999). Research studies have revealed that positive psychological traits can alleviate the negative impact of work–family conflict on individuals, such as resilience (Bernuzzi et al., 2021), emotional intelligence (Sharma et al., 2016), and self-efficacy (Wattoo et al., 2020). Self-efficacy is the overall self–confidence of individuals when dealing with different environments and pressure challenges (Schwarzer and Born, 1997). Self-efficacy theory points out that individuals with high self-efficacy are more likely to believe that they have the ability to deal with passive stressors, and are less likely to regard these stressors as threats (Bandura, 1994). Previous research studies have indicated that individuals with high self-efficacy were better at dealing with conflict events and appeared to have a lower level of emotional exhaustion (Skaalvik and Skaalvik, 2010; Yu et al., 2015). On the contrary, individuals with low self-efficacy were more likely to worry about their insufficient resources to deal with conflict events, and more likely to feel resource depletion and to behave listlessly (Hall et al., 2019). Based on these, we established our third hypothesis:

**Hypothesis 3:** Self-efficacy plays a moderating role in the direct path and the second half of the mediating path.

**FIGURE 1:**
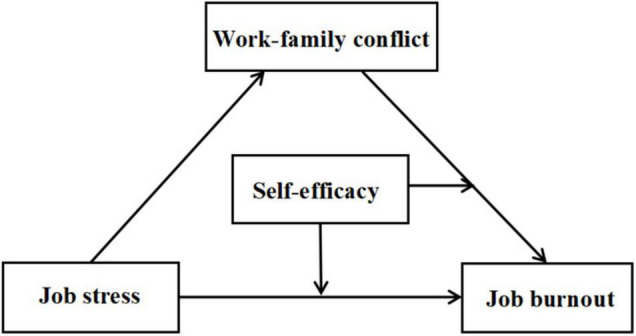
The proposed moderated mediation model.

In summary, this study constructed a model (as shown in Figure 1) to explore the mediating and moderating mechanisms of job stress predicting the teacher burnout, to provide ideas for preventing and relieving teacher burnout. Three hypotheses were put forward: (1) job stress has a significant predictive effect on job burnout; (2) work–family conflict plays a mediating role in the relationship between job stress and job burnout; and (3) self-efficacy plays a moderating role in the direct path and the second half of the mediating path.

## Materials and Methods

### Participants

The participants included 600 primary and secondary teachers from Shandong province, China. Cluster sampling was adopted for the research objects, who were selected from five primary and secondary schools. After excluding invalid questionnaires with incomplete answers, 558 valid questionnaires were obtained (valid recovery rate = 93%). Among the valid samples, there were 171 men (30.6%) and 387 women (69.4%), ranging in age from 19 to 60 years. The average age of participants was 35.44 years (SD = 9.42), 409 (73.3%) were married, and 149 (26.7%) were unmarried; 39 (7%) had a college degree, 467 (83.8%) had a bachelor’s degree, and 51 (9.2%) had a master’s degree; 218 were head teachers (39.1%) and 340 non-head teachers (60.9%). The years of teaching experience ranged from 1 to 40 years, including 217 (38.9%) with 1–5 years, 71 (12.7%) with 6–10 years, 121 (21.7%) with 11–20 years, and 149 (26.7%) with over 21 years of teaching experience. The average number of years of teaching experience was 16.18 years (SD = 9.08).

### Measures

#### Job Stress Scale of Teachers

The job stress scale of primary and secondary school teachers compiled by Zhu et al. (2002) was used to measure the source and intensity of job stress among primary and secondary school teachers. A total of 46 items were scored on a 5-point scale (1 = “no pressure,” 5 = “great pressure”). The scale was divided into six dimensions: examination stress, stress related to student factors, stress in self-development needs, interpersonal stress, stress in workload, and stress in career expectations. Responses to all items were averaged, with higher scores indicating higher levels of job stress. In this study, the Cronbach’s alpha coefficient of the scale is 0.97. Amos 22.0 software was used to conduct the confirmatory factor analysis (CFA). The results are as follows: χ^2^/*df* = 3.616, GFI = 0.763, NFI = 0.835, TLI = 0.865, CFI = 0.874, and RMSEA = 0.069.

#### Teachers’ Burnout Questionnaire

The Chinese Primary and Secondary School Teachers’ Job Burnout Questionnaire was revised by Wu et al. (2016) on the basis of the Maslach’s Burnout Inventory–Educators Survey (MBI–ES). The scale was divided into three dimensions: emotional exhaustion, depersonalization, and reduced personal accomplishment, with a total of 22 items. The items were scored on a 7-point scale (0 = “never,” 6 = “every day”). Responses to all items were averaged, with higher scores indicating higher levels of job burnout. In this study, the Cronbach’s alpha coefficient of the scale is 0.87. Amos 22.0 was used to conduct the CFA. The results are as follows: χ^2^/*df* = 3.616, GFI = 0.763, NFI = 0.835, TLI = 0.865, CFI = 0.874, and RMSEA = 0.069, indicating that the structural validity of the scale is acceptable.

#### Work–Family Conflict Questionnaire

The work–family conflict questionnaire of primary and secondary school teachers was compiled by Wu et al. (2009). The 5-point (1 = “rarely,” 5 = “always”) scale, including 22 items, is divided into two dimensions: work interfering with family (WIF) and family interfering with work (FIW). Responses to all items were averaged, with higher scores indicating higher levels of work–family conflict. In this study, the Cronbach’s alpha coefficient of the scale is 0.964. The internal consistency reliability of work interfering WIF and FIW subscales are 0.945 and 0.943, respectively. Amos 22.0 was used to conduct the CFA. The results are as follows: χ^2^*/df* = 4.265, GFI = 0.895, NFI = 0.942, TLI = 0.935, CFI = 0.954, and RMSEA = 0.077, indicating that the structural validity of the scale is acceptable.

#### Self-Efficacy Scale

The general self-efficacy scale (GSES) was compiled by Schwarzer and Born (1997). The 5-point scale (1 = “totally disapproval,” 5 = “totally approve”) includes 10 items. Responses to all items were averaged, with higher scores indicating higher levels of self-efficacy. In this study, the internal consistency reliability of the scale is 0.942. Amos 22.0 was used to conduct the CFA. The results are as follows: χ*^2^*/*df* = 3.444, GFI = 0.977, NFI = 0.986, TLI = 0.976, CFI = 0.990, and RMSEA = 0.066, indicating that the structural validity of the scale is good.

#### Control Variables

According to previous studies, teachers of different gender, years of teaching experience, and marital status may have different job burnout. These resources directly affect teachers work experience of job roles and their level of stress (Luk et al., 2010; Ma et al., 2021). Therefore, gender, age, and education were used as control variables in the current study.

### Procedure

This study used a paper questionnaire administered to teachers from five primary and secondary schools in Shandong province. The main test was administered by professionally trained graduate students of psychology. The questionnaire guidelines emphasized the authenticity of the answers and the anonymity of the survey, and the questionnaire was completed by the teachers separately. It took about 15 min to complete all the questionnaires. This study obtained the informed consent of the teachers, and was approved by the ethics committee of the author’s institution and the investigated primary and secondary schools.

### Data Analysis

SPSS version 22.0 (IBM, NY, United States) was used for the statistical analysis. Descriptive statistics were produced for all variables, while the PROCESS macro for SPSS (Model 4) was applied to examine the mediating effect of work–family conflict. Finally, the PROCESS macro for SPSS (Model 15) was used to examine the moderated mediating effect of self-efficacy on the direct path and the second half of the mediating path (Hayes, 2013). In the current study, missing data were handled *via* the maximum likelihood estimates (ML).

## Results

### Common Method Deviation Test

As all the survey data were from the teacher self-reports, there may be common method deviation. Therefore, the Harman single factor test was used to test the deviation of variables. The results showed that the eigenvalues of 14 factors were greater than 1, and the explanatory power of the first factor was less than 40% of the critical value (the value of variation was 28.87%). Therefore, common method bias did not affect the data results.

### Preliminary Analysis

The descriptive statistical results are shown in Table 1. The results showed that teacher job stress was positively correlated with work–family conflict (*r* = 0.60, *p* < 0.001) and job burnout (*r* = 0.72, *p* < 0.001), and work–family conflict was positively correlated with job burnout (*r* = 0.47, *p* < 0.001). In addition, self-efficacy was negatively correlated with job burnout (*r* = −0.18, *p* < 0.001). A *t*-test was conducted to assess whether there were gender differences between the following variables. The results showed that there were significant gender differences in job stress and self-efficacy (*t* = 3.49; *p* < 0.01; *t* = 2.48; *p* < 0.05). Compared with male teachers, female teachers had more job stress and lower self-efficacy. However, there was no significant gender difference in work–family conflict and teachers’ job burnout (*t* = 0.65; *p* = 0.52; *t* = 1.26; *p* = 0.21).

**TABLE 1:** Descriptive statistics and correlation among variables (*N* = 558).

	1	2	3	4	5	6	7
1. Gender	1						
2. YTE	−0.37***	1					
3. Marriage	0.18***	−0.58***	1				
4. Job stress	−0.16***	0.15**	−0.10*	1			
5. WFC	−0.03	0.04	−0.08	0.60***	1		
6. Job burnout	−0.05	−0.03	−0.01	0.45***	0.47***	1	
7. Self-efficacy	−0.11*	0.11*	0.01	0.00	−0.06	−0.18***	1
*M*	1.69	12.48	1.27	2.91	2.11	1.93	3.18
SD	0.46	10.54	0.44	0.84	0.86	0.90	0.87

*WFC, work–family conflict; YTE, years of teaching experience.*

*p < 0.05, **p < 0.01, and ***p < 0.001.

### Mediating Effect Analysis

Model 4 of the PROCESS macro was used to investigate the predictive effect of teacher job stress on burnout, and the mediating role of work–family conflict (Hayes, 2013). As Table 2 shows, job stress was positively associated with work–family conflict (β = 0.61, *t* = 17.84, *p* < 0.001), which in turn was positively related to teacher burnout (β = 0.30, *t* = 6.65, *p* < 0.001). The positive direct association between job stress and burnout perpetration remained significant (β = 0.27, *t* = 5.85, *p* < 0.001). Therefore, Hypothesis 1 was supported. Work–family conflict partially mediated the relationship between job stress and burnout (indirect effect = 0.19, SE = 0.03, 95% CI = [0.13, 0.52]). The mediation effect accounts for 41% of the total effect of job stress on job burnout.

**TABLE 2:** Testing the mediation effect of job stress on burnout.

Predictors	Model 1 (job burnout)		Model 2 (WFC)		Model 3 (job burnout)	
	β	*t*	β	*t*	β	*t*
Gender	–0.02	–0.46	0.06	1.64	–0.04	–0.94
YTE	−0.12*	–2.49	–0.07	–1.54	−0.10*	–2.15
Marriage	–0.03	–0.57	–0.07	–1.63	–0.01	–0.13
Job stress	0.46***	11.93	0.61***	17.84	0.27***	5.85
WFC					0.30***	6.65
*R* ^2^	0.21		0.37		0.27	
*F*	138.90***		314.04***		97.88***	

*WFC, work–family conflict; YTE, years of teaching experience.*

*p < 0.05 and ***p < 0.001.

### Moderated Mediation Effect Analysis

**FIGURE 2:**
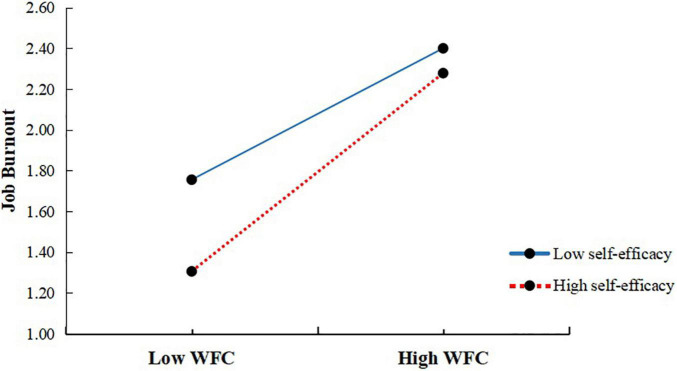
Interaction between work–family conflict and self-efficacy on job burnout.

To test the moderated mediation model, we used Model 15 of the SPSS PROCESS macro compiled by Hayes (2013). After controlling for gender, years of teaching experience, and marriage, the self-efficacy moderation test was conducted; the results are shown in Table 3. As shown in the model (Burnout), the product (interaction term) of work–family conflict and self-efficacy had a significant predictive effect on burnout (β = 0.09, *t* = 1.97, *p* < 0.05), and the effect of the product (interaction term) of job stress and self-efficacy was not significant (β = 0.01, *t* = 0.09, *p* > 0.05). Therefore, we plotted predicted work–family conflict against burnout, separately for low- and high-levels of self-efficacy (*M* ± 1 SD). Simple slope tests showed that for teachers with high self-efficacy, work–family conflict significantly predicted burnout, β*_*simple*_* = 0.57, *t* = 6.99, *p* < 0.001. However, for teachers with low self-efficacy, work–family conflict significantly predicted burnout but to a much weaker extent, β*_*simple*_* = 0.38, *t* = 6.99, *p* < 0.001 (Figure 2). The results showed that with the increase of self-efficacy of teachers, the predictive effect of work–family conflict on burnout gradually increased.

**TABLE 3:** Testing the moderated mediation effect of job stress on burnout.

Predictors	*R* ^2^	*F*	β	*t*
Model (job burnout)	0.3	29.68***		
Gender			–0.09	–1.18
YTE			–0.007	–1.72
Marriage			0.02	0.17
Job stress			0.29	5.82***
Self-efficacy			–0.17	−4.49***
WFC × self-efficacy			0.09	1.97*
Job stress × self-efficacy			0.01	0.09

*WFC, work–family conflict; YTE, years of teaching experience.*

*p < 0.05 and ***p < 0.001.

## Discussion

Based on the conservation of resources theory, this study investigated the current situation of the occupational stress experienced by the Chinese primary and secondary school teachers in this critical period of Chinese basic education reform. It has confirmed a relationship between the occupational stress and job burnout of the Chinese primary and secondary school teachers, and provides strong support for the applicability of the conservation of resources theory to the Chinese context. Moreover, due to the consideration of the Chinese “family standard” culture, this study first selected the variable “work–family conflict” to prove its mediating role between job stress and burnout. This fills a research gap by confirming the mediating mechanism between teachers’ job stress and burnout in the context of the Chinese culture. More importantly, different from previous findings, this study found that the protective effect of self-efficacy in mediating between work–family conflict and burnout is limited; that is, among the teachers with high self-efficacy, work–family conflict had a stronger predictive effect on job burnout. This finding suggests that self-efficacy may play a more complex role in the mechanism of job burnout, and its protective effect on burnout may change in the context of stress and conflict.

The results showed that job stress had a significant positive predictive effect on job burnout. Research Hypothesis 1 was verified, which is consistent with previous research results (Kim and Kweon, 2020). On the one hand, primary and secondary school teachers have to face numerous requirements from schools, colleagues, student management, society, and families of students. When they think that the available resources and abilities do not meet the job requirements, they will face professional pressure, resulting in a series of burnout behaviors. On the other hand, it is difficult for primary and secondary school teachers to get a considerable return on a large amount of work investment. The imbalance between work investment and material and spiritual returns easily results in the emotional exhaustion and decreased work enthusiasm for teachers (Zhou and Li, 2021), which results in the negative emotion of job burnout.

### The Mediating Role of Work–Family Conflict

This study found that work–family conflict played a mediating role between the job stress and job burnout of teachers. Excessive job stress will aggravate work–family conflict, and then affect the job burnout of teachers. This study emphasizes the important role of family in influencing the job burnout of teachers. When conflict between work and family is difficult to reconcile, it will affect the development of individuals and organizations (Kreiner et al., 2009).

On the one hand, the requirements from students, society, and school evaluation make teachers perceive that they have an excessive number of tasks in their workload or that they do not have enough time and energy to complete these tasks, resulting in a lot of work pressure (Li et al., 2011). At this time, if the emotional needs of family members (partners and children) are not met and they feel neglected, they may produce more negative emotions, which will exacerbate family conflict (Kang et al., 2020). On the other hand, according to the conservation of resources theory, when teachers are in a state of work–family conflict, their psychological resources are largely invested in maintaining the coordination and balance of work and family relations (LePine et al., 2004). If the consumed psychological or emotional resources are not supplemented in time, it is likely to affect the emotional and psychological resources invested by teachers in their work, which leads to serious job burnout.

### The Moderating Role of Self-Efficacy

The results of the moderating effect analysis showed that self-efficacy played a significant moderating role in the path of work–family conflict to job burnout, which partially verified Hypothesis 3. In addition, this study found that for teachers with high self-efficacy, although the degree of job burnout was lower than teachers with low self-efficacy, the predictive effect of work–family conflict on job burnout was stronger. Previous studies have shown that if individuals have a high sense of self-efficacy, they are more likely to mobilize their own motivation and cognitive resources to coordinate and balance these conflicts in the face of work–family conflicts (Deuling and Burns, 2017), but this result is to the contrary. This may be because individuals with high self-efficacy expect themselves to maintain a good image in their work and life, and are more confident in their ability to control and deal with work–family conflict (Wattoo et al., 2020). However, some work–family conflicts may be caused by more serious or unusual events (such as, the serious illness of family members and problems of supporting the elderly), which are usually considered uncontrollable. At such times, individuals with high self-efficacy may feel depressed because they have tried to control these uncontrollable events but failed (Glaser and Hecht, 2013), while individuals with low self-efficacy are more used to accept that some things are uncontrollable. Therefore, teachers with high self-efficacy may experience more serious job burnout in the face of work–family conflict.

In addition, this study tested the moderating effect of self-efficacy on job stress and job burnout of teachers. The results showed that the moderating effect was not significant. This may be related to the fact that most of the participants in this study were women. Affected by uneven social employment resources and the Chinese traditional culture where men are held superior to women, the self-efficacy levels of women were generally lower than those of men (Xie et al., 2016). Previous studies have shown that the relationship between self-efficacy and positive and negative factors of mental health is weaker in female groups (Li et al., 2019). Because of the generally low-level of self-efficacy (there may be a floor effect) and the fact that the relationship between self-efficacy and mental health factors is weak in female groups, the moderating effect of self-efficacy between job stress and job burnout may be very limited in this study. We should therefore pay more attention to the mental health of female teachers and promote the continuous improvement of self-efficacy of female teachers.

### Practical Significance and Limitations

The current research has the following crucial theoretical and practical contributions. First, it underlines the importance of creating a comfortable professional environment, improving the evaluation system of primary and secondary school teachers in China, and enhancing the positive emotional experience of teachers to alleviate job stress of teachers. Second, it is important for teachers to pay more attention to the position of their family in their work and lives, to maintain a balance between work and family, and strengthen the communication with family members. Additionally, schools should formulate a reasonable work schedule so that teachers can have sufficient rest time to have good relations with their families, so as to reduce the conflict between work and family. Finally, this study revealed that self-efficacy has a complex role in work–family conflict, highlighting that individuals with high self-efficacy need to distinguish between the types of work–family conflict in their daily life, and adopt more gentle ways to deal with uncontrollable conflicts to avoid aggravating emotional exhaustion.

Several limitations need to be considered when interpreting the findings. First, our cross-sectional data limit causal inferences. Previous studies have also indicated that work–family conflict can contribute to job stress (Liu et al., 2017; Mack and Rhineberger-Dunn, 2019). Therefore, it is necessary to use longitudinal designs to obtain stronger empirical evidence of causal evidence in future research. Second, this study only considered the negative effects of job stress and ignored the positive effects of some challenging stressors (such as, time urgency and high sense of responsibility associated with work) (Cavanaugh et al., 2000; Zhou et al., 2021). Future research should conduct a comprehensive analysis of the different nature of stressors, adopting a dialectical approach to consider the role of job stress on the job burnout of teachers. Finally, although this study tested the moderating role of self-efficacy between work–family conflict and job burnout, future research needs to conduct a more in-depth study on the complex mechanism of self-efficacy on burnout, to promote the healthy development of mental health of teachers.

## Conclusion

In summary, this study is an important step forward in understanding how job stress relates to the job burnout of Chinese teachers. It has very important significance for the Chinese primary and secondary school teachers who want to alleviate their professional pressure and reduce their level of job burnout. First, it reveals the relationship between job stress and burnout of primary and secondary school teachers in the critical period of the Chinese basic education reform. Second, it shows that work–family conflict serves as a mediating role between job stress and burnout, which highlights the important role of family in the job stress and burnout of teachers. Moreover, the relationship between work–family conflict and job burnout is moderated by self-efficacy, and the relationship appears to be stronger for teachers with high self-efficacy than for those with low self-efficacy. It shows that self-efficacy may not be able to alleviate teacher burnout caused by work–family conflict as expected.

## Data Availability Statement

The original contributions presented in the study are included in the article/supplementary material, further inquiries can be directed to the corresponding author.

## Ethics Statement

This study was carried out in accordance with the recommendations of the Institutional Review Board of Shandong Normal University with written informed consent from all subjects in accordance with the Declaration of Helsinki. The protocol was approved by the Institutional Review Board of Shandong Normal University. The patients/participants provided their written informed consent to participate in this study. Written informed consent was obtained from the individual(s) for the publication of any potentially identifiable images or data included in this article.

## Author Contributions

WZ was the principal investigator. XL and QL collected and analyzed the data under the supervision of WZ. WZ and XL designed the study and contributed to materials and analysis tools. WZ, XL, WJ, and WD contributed to the writing of the manuscript. WZ and WD provided guidance to the manuscript. All authors contributed to the article and approved the submitted version.

## Conflict of Interest

The authors declare that the research was conducted in the absence of any commercial or financial relationships that could be construed as a potential conflict of interest.

## Publisher’s Note

All claims expressed in this article are solely those of the authors and do not necessarily represent those of their affiliated organizations, or those of the publisher, the editors and the reviewers. Any product that may be evaluated in this article, or claim that may be made by its manufacturer, is not guaranteed or endorsed by the publisher.
